# Nasally inhaled therapeutics and vaccination for COVID‐19: Developments and challenges

**DOI:** 10.1002/mco2.101

**Published:** 2021-12-14

**Authors:** Jinxiang Xi, Lameng Ray Lei, William Zouzas, Xiuhua April Si

**Affiliations:** ^1^ Department of Biomedical Engineering University of Massachusetts Lowell Massachusetts USA; ^2^ Amphastar Pharmaceuticals, Inc Rancho Cucamonga California USA; ^3^ Department of Aerospace Industrial and Mechanical Engineering California Baptist University Riverside California USA

**Keywords:** ACE2, COVID‐19 management, inhaled vaccine, mucociliary clearance, nasal spray, viral entry

## Abstract

The nose is the initial site of viral infection, replication, and transmission in the human body. Nasally inhaled vaccines may act as a promising alternative for COVID‐19 management in addition to intramuscular vaccination. In this review, the latest developments of nasal sprays either as repurposed or antiviral formulations were presented. Nasal vaccines based on traditional medicines, such as grapefruit seed extract, algae‐isolated carrageenan, and Yogurt‐fermenting *Lactobacillus*, are promising and under active investigations. Inherent challenges that hinder effective intranasal delivery were discussed in detail, which included nasal device issues and human nose physiological complexities. We examined factors related to nasal spray administration, including the nasal angiotensin I converting enzyme 2 (ACE2) locations as the delivery target, nasal devices, medication translocation after application, delivery methods, safety issues, and other nasal delivery options. The effects of human factors on nasal spray efficacy, such as nasal physiology, disease‐induced physiological modifications, intersubject variability, and mucociliary clearance, were also examined. Finally, the potential impact of nasal vaccines on COVID‐19 management in the developing world was discussed. It is concluded that effective delivery of nasal sprays to ACE2‐rich regions is urgently needed, especially in the context that new variants may become unresponsive to current vaccines and more refractory to existing therapies.

## Graphical abstract

Nasal spray vaccines and therapeutics can be a potent alternative for COVID‐19 management. The latest developments of nasal sprays either as repurposed or antiviral formulations were presented. Inherent challenges that hinder effective intranasal delivery were discussed in detail, which included nasal device issues and human nose physiological complexities. The need to develop an effective delivery system to target formulations at the ACE2‐rich regions is particularly pressing in the context of emerging SARS‐CoV‐2 variants that evade current vaccines and develop resistance to existing therapies.

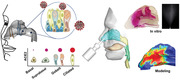

## 1 INTRODUCTION

### 1.1 Advantages of nasally inhaled therapeutics and vaccination for COVID‐19

COVID‐19 is a highly transmissible respiratory infectious disease due to the SARS‐CoV‐2 virus. Up to the moment (October 15, 2021), it has caused 239,437,517 confirmed cases and 4,879,235 deaths, while a total of 6,495,672,032 vaccine doses have been administered.^1^ It has been proven to be highly challenging to prevent viral transmission because of its high transmissibility and constantly evolving variants. Vaccines and therapeutics have been actively developed since the pandemic all over the world. However, there are only a handful of vaccines that have been approved or via emergence use authorization (EUA) for vaccination/treatment of COVID‐19, all of which are intravenously administered.^2^ There exists a pressing need to develop inhalation‐based vaccines/therapies to assist COVID‐19 management, which should take into account the infection onset (viral entry), progression (viral shedding), and exacerbation, where quick viral replication and cytokine storms in the pulmonary alveoli lead to pneumonia and respiratory failure.^3^

Human nasal passages are the initial dominant sites of viral invasion, replication, and transmission. Nasal sprays can act as a potent alternative for COVID‐19 therapeutics and vaccination. Spray formulations that can either inactivate SARS‐CoV‐2 or block its entry into cells will be promising to decrease viral load in the nose, thus preventing the viral spread to the lung or surrounding people. Vaccines administered through the nasal route will trigger the releasing of immunoglobulins, mucosal IgA, and serum IgG, both of which have been shown to increase the vaccination efficacy.^4^ Furthermore, nasal mucosa vaccination can enhance cross‐protection through the generation of cross‐reactive antibodies.^5^

Nasally inhaled vaccines offer multiple advantages in comparison to traditional routes, such as intramuscular and oral routes. It is safe, easy to apply, and can induce both topical and systemic immune responses.^6^ Inhaled vaccines administered as the nasal spray is needle‐free; most of them are low‐cost, do not require refrigeration for storage and shipping, and are amenable for self‐administration, thus reducing the demand on healthcare staff and facility.

### 1.2 Nasal spray COVID‐19 management: a solution for low‐income countries?

The socioeconomic status of a community, city, or country has been proven to affect COVID‐19 incidence and mortality in that group.^7^ In developing countries, the pandemic has adversely impacted the population with low socioeconomic status the most, due to crowded living conditions, insufficient health care, inadequate testing and vaccination, and limited resources to work remotely. Mortality rates are higher in developing countries and low‐income neighborhoods. Due to healthcare facility inequalities, 90% of COVID‐19‐related deaths in lower‐income areas happened outside of the hospital in Santiago Chile, in comparison to 55% in the hospital in more affluent areas.^7^ Up to date (October 15, 2021), only 2.7% of people in low‐income countries have received at least one dose, in contrast to 47.4% worldwide.^2^

**FIGURE 1:**
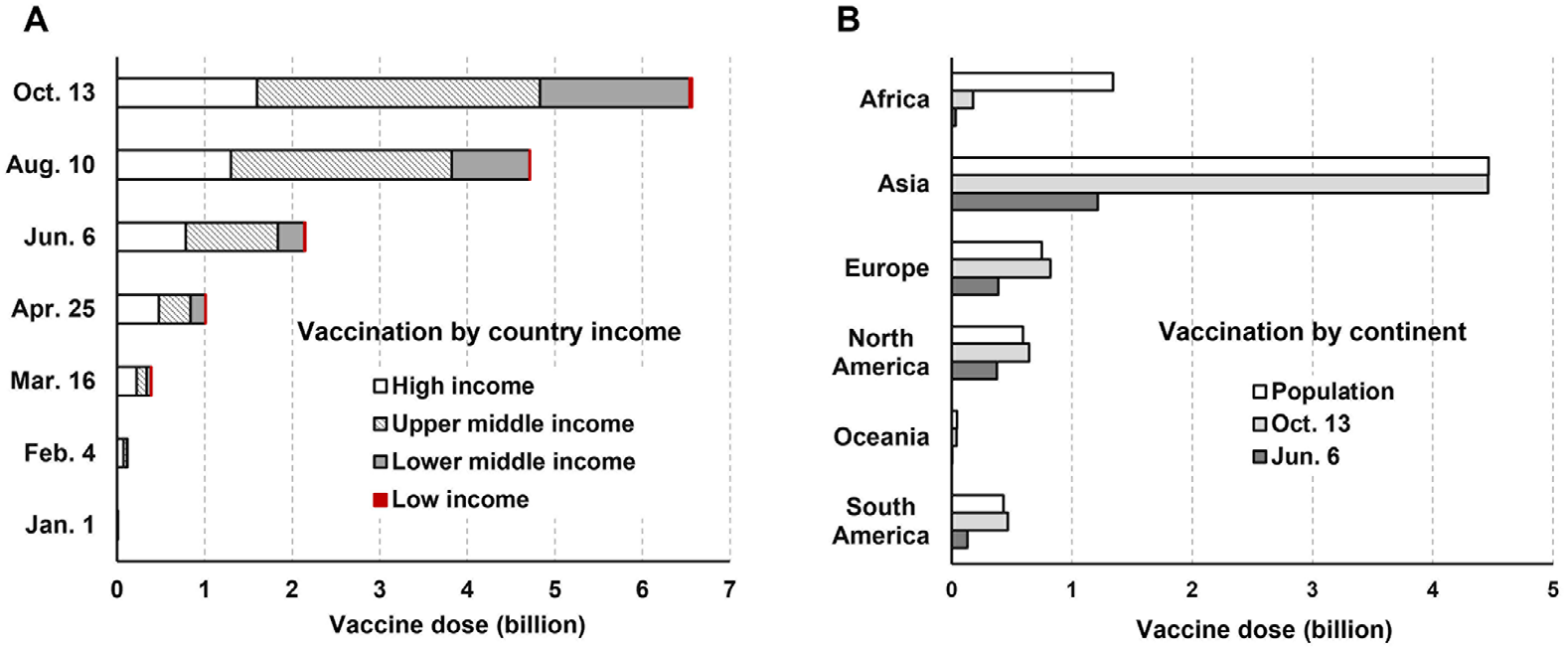
COVID‐19 vaccination: (A) by country income and (B) by continent (data source: ourworldindata.org, October 15, 2021)

People who have been infected by coronavirus will display symptoms within 2–14 days and contact tracing is impractical in most countries. Additionally, many infected individuals are asymptomatic. Not knowing that one is sick will encourage the individual to continue going out and not abiding by preventive measures, which can exponentially increase the viral infectivity.^8^ This problem can be more pronounced in the developing world, where the population density is large and social distancing is more difficult. Both the vaccine doses and the vaccination rate in lower‐middle‐income countries and low‐income countries are strikingly lower than those in high‐income countries (Figure 1A). Despite that 22.4 million vaccination doses were administered each day worldwide, there is only a 2.6% vaccination rate in Africa by June 03 and a 13.2% vaccination rate by October 15 (Figure 1B).^2^ Facing a shortage of vaccines and healthcare facilities, intranasal inhalation therapy can be a viable alternative to prevent COVID‐19 infection as the first‐line barrier to both viral entry and viral shedding. Nasal sprays with a nitric oxide releasing solution (NORS) or a calcium‐rich saline formulation may halt viral entry and decrease exhalation of respiratory droplets, both of which can help contain community viral transmissions.^9^, ^10^

In hope that nasal spray vaccines for COVID‐19 will become available soon, they will provide low‐cost alternatives for COVID‐19 control in the developing world. A nasal spray therapy is well suited for self‐administration by the patients, lowering the hospital visit demand, and thus easing the burden of the healthcare facility, as well as eliminating the chances of cross‐exposures. The needle‐free feature of nasal sprays also makes it more appealing to young children and their parents. Up to date, it is still not clear whether the low COVID‐19 mortality rate in children and young adults is because of a lower infection rate or a stronger immune system. Considering the latter scenario, children can act unknowingly as viral sources and pose a threat to their peers and older relatives. A frequent nasal spray application may effectively diminish this risk.

In response to the shortage of vaccines and purported therapeutics, many efforts are actively undertaken to explore low‐cost, readily available materials that can boost immunity or cure symptoms. It is desirable that the new vaccines or drugs can be produced using existing worldwide infrastructure, that is, for seasonal flu viruses, or for other respiratory diseases, such as asthma, or chronic obstructive pulmonary disease (COPD). For instance, *Lactobacillus*, a bacteria widely used in probiotic supplements with no known safety issues, has been explored as a vaccine vector to develop a nasal spray vaccine for COVID‐19, which can colonize the respiratory mucosa in harmony with other bacteria lining the airway and provide a longer protection time.^11^ *Lactobacillus* is inexpensive to prepare and ready for genetic modification, thereby reducing the cost of antigen purification. Considering the severe shortage of vaccines in most developing countries, this and similar others can be valuable in fighting against the SARS‐CoV‐2 and its variants, which are still ravaging the world.

### 1.3 Outline

In this review, we will first briefly explain the infection mechanisms of COVID‐19 in Section 2, then present the latest developments of nasally inhaled therapeutics and vaccines for COVID‐19 management in Section 3. The devices for nasal administration and the challenges associated with current devices will be discussed in detail in Section 4. This will be followed by the discussion of nasal physiological factors that affect the distribution of nasally inhaled medications in Section 5. We hope that through this review, the readers can have a better understanding of the latest developments of nasally inhaled vaccines and treatments for COVID‐19 management, as well as the challenges that may hinder the effective delivery to the target tissues in the nose for optimal outcomes.

## 2 COVID‐19 INFECTION MECHANISMS

### 2.1 Viral entry to airway cells

The SARS‐CoV‐2 viruses were found to range between 60 and 140 nm in diameter and could be conveyed in droplets and particles of a wide range.^12^ There are three major phases of infection for COVID‐19. The first phase is characterized by viral replication. Coronavirus enters the respiratory tract through the mouth or nose. Once deposited on the airway epithelium, the virus enters the cells through spike proteins found on the surface of the virus. The spike (S) protein of the coronavirus functions by recognizing receptors on host cells and mediating a membrane fusion process.^13^ The SARS‐Cov‐2 virus invades two types of cells in the respiratory tract: goblet cells and ciliated cells.^14^ Once the host cells have been infected, they are used by the coronavirus for viral replication. When these cells die, they fall off and fill the respiratory tract with debris and fluid. This is when the symptoms of cough, fever, and difficulty breathing appear. The human body then reacts to the pathogens through the onset of the immune system. This will lead to the second phase of infection, which is characterized by the hyper‐reactivity of the immune system. During this phase of infection, the hyperactivated immune system can develop a cytokine storm that damages body tissues.^15^ The hyperinflammatory response leads to the third phase of infection, with progressive destructions of pulmonary tissues. These include the development of holes in the lungs, the increased permeability of the alveoli, and the accumulation of fluids inside the lungs. Patients who experience pulmonary destruction often need the use of mechanical ventilation to facilitate breathing.

### 2.2 Viral activities in nasal mucosa

The human nasal passages are the main gateway for the coronavirus. The nasal mucosa is one of the major sites with quick viral replications and has been used widely as the sampling site (i.e., nasal swab) in COVID‐19 testing. Once the virus‐laden droplets are deposited on the nasal mucosa, viral invasion and replication start in the ciliated epithelial cells over a 3‐ to 14‐day period with no apparent symptoms. After that, an inflammatory response similar to the common cold manifests and damages the epithelium cells. Replicated viruses are shed in the nasal secretions and enter the lower respiratory tract as debris, which may catalyze a hypersensitive immune response in the alveoli (i.e., cytokine storm), leading to pneumonia, low oxygen rate, respiratory failure, and even death.

**FIGURE 2:**
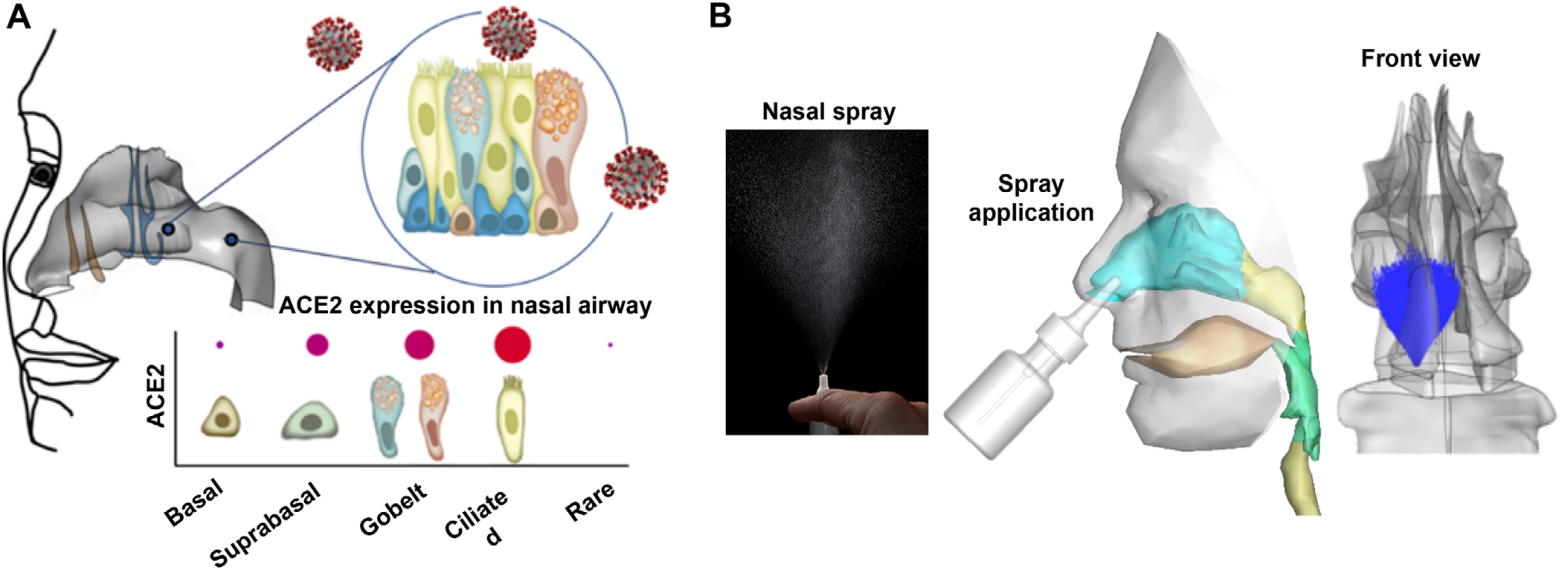
Nose as the initial site for viral invasion and therapy: (A) ACE2 expression in the nasal cavity and (B) nasal physiology that presents challenges to effective nasal spray delivery

The process of host cellular penetration begins with the contact between the viral spike's receptor‐binding domain (RBD) and the angiotensin I converting enzyme 2 (ACE2) receptor on the human cell surface (Figure 2A).^13^ Next, the host's type II transmembrane protease (TMPRSS2) breaks the spike protein twice: once between the S1 and S2 subunits and once within the S2 subunit. This crucial cleavage step, known as priming, allows the binding of the spike protein to the cell membrane. Inside the cell, the virus hijacks host cell transcription and quickly replicates itself. Finally, matured viruses are released from the infected cells to infect other cells. Due to the pivotal role of S protein in COVID‐19 infection, the spike receptor has been the biological target for COVID‐19 therapy and vaccine development.

Other host receptors besides ACE2 have been discovered to participate in facilitating viral entry. Recent studies identified that neuropilin‐1 (NRP1), a protein capable of binding furin‐cleaved substrates, interacts with S protein directly and that this effect was reversed by mutation of the furin cleavage site.^16^, ^17^ Notably, the spike protein of SARS‐CoV‐2 does not contain a furin cleavage site. Thus, the abundant expression of NRP1 in respiratory and olfactory epithelia could help explain the more virulent nature of SARS‐CoV‐2 compared to SARS‐CoV. In early 2021, yet another receptor, tyrosine‐protein kinase receptor UFO (AXL), was discovered to interact with S protein and promote infectivity.^18^ AXL binds to the S‐protein's N terminal domain (NTD), whereas NRP1 binds to the Cend rule (CendR) motif, which is at the N‐ and C‐terminus of the S protein's S1 subunit, respectively.^18^

## 3 NASAL SPRAY DEVELOPMENT FOR COVID‐19 MANAGEMENT

### 3.1 Prevention and therapeutics

The primary means of SARS‐CoV‐2 transmission was through aerosols and droplets, and the nose is the initial dominant site of viral infection, replication, and transmission. While hand sanitizing has become the norm, protecting and cleaning the nose should become routine too.^19^ The nose, together with the mouth, is responsible for producing aerosols and droplets that may infect others.^20^ Community‐based inhalation therapy using the nasal spray is commendable to reduce viral load in the nose and minimizing exhaled virus‐laden droplets; such therapy is not meant to be a replacement to the vaccine program, but as a mitigation measure for SARS‐CoV‐2 and its new variants.

Recent clinical trials have shown that nasal sprays with calcium chloride or nitride oxide (NO) can be an effective and safe antiviral therapy to inhibit COVID‐19 infection and decrease symptom severity. Edwards et al.^10^, ^21^ proposed a nasal calcium‐rich saline formulation to decrease the exhalation of respiratory droplets. This drug‐free solution interacts with the airway lining through the two positive charges of the calcium ion binding with negatively charged mucus proteins. The results showed a 75% decrease of expiratory droplets in a cohort of 76 subjects, making one less infectious to others and acting as a form of source control. Burton et al. proposed the use of NORS as a nasal spray to treat mild and moderate COVID‐19 patients.^9^ NORS can release a concentration of NO for a sustained duration that is high enough to neutralize the SARS‐CoV‐2 virus. Israel and New Zealand have issued interim approval for NORS nasal spray. The function of NO in killing the virus, blocking viral entry into cells, and halting viral replication has been demonstrated and thus is promising to rapidly reduce viral load within the nasal passages in the form of nasal spray.^22^ It is also noted that the safety for NO clinical usages in newborn babies as well as adults has been established for decades.^23^

Xiang et al. investigated *Lactobacillus*, a bacteria used in yogurt fermentation, as the antigen to be directly delivered to nasal mucosa using nasal spray, which is hypothesized to elicit the immune responses right at the location of viral entry and replication.^11^ This targeted, site‐specific intervention is expected to give even improved protection against COVID‐19 than vaccines given intramuscularly, as it is more like the natural viral infection, generating antibodies and immune responses, where the SARS‐CoV‐2 virus enters.^11^

### 3.2 Vaccination

Since the pandemic, COVID‐19 vaccines have been developed at an unprecedented speed.^24^ According to the World Coronavirus Vaccine Tracker and the COVID‐19 Vaccines FDA up to October 15, 2021, 105 vaccines are undergoing human clinical trials, 35 vaccines are at the final testing stage, and at least 75 preclinical vaccines are being actively investigated using animal tests.^25^, ^26^ Among them, nine vaccines have been approved for use. These include Pfizer‐BioNTech, Moderna, and Johnson & Johnson in the United States and European Union, CanSino, Sinovac, and Sinopahrm‐Wuhan in China, AstraZeneca in Brazil, Sputnik‐V in Russia, and EpiVacCorona in Turkmenistan.^25^, ^26^ Recently (May 10, 2021), the FDA expanded theEUA of the Pfizer‐BioNTech vaccine to be used in young people aged 12 through 15.^26^

All the above nine vaccines are administered intramuscularly to the upper arm. On the other hand, the delivery of vaccines as a nasal spray is getting more attention. There are at least five nasal spray vaccines at the phase 1 clinical trial. These include (1) Mambisa in Cuba that contains a coronavirus spike protein RBD from November 2020,^25^ (2) Oxford‐AstraZeneca that is tailored to the B.1.351 variant from February 2021,^25^ (3) the DelNS1‐LAIVs (Deletion of Nonstructural Protein 1 and live attenuated influenza virus) vaccine in China from March 2021,^27^ (4) Maryland‐based AdCOVID with Ad5 adenovirus from December 2020, and (5) BBV154 in India that contains a chimpanzee adenovirus from February 2021.^28^ The Cov‐Pars Razi vaccine in Iran that contains fragments of coronavirus spike proteins and is delivered as a nasal spray has finished the phase I trial and will enter phase II from April 2021.^25^ All six nasal spray formulations have the COVID‐19 spike protein and will be targeted at the nasal ciliated epithelium, and thus can be more effective for inhibiting viral activities than intramuscularly given vaccines.^29^

Up to date, no inhaled COVID‐19 vaccine has been approved. The first clinical trial result of the inhaled COVID‐19 vaccine was reported by Wu et al., who evaluated the immunogenicity and safety of the Ad5‐nCoV (adenovirus type‐5 vector‐based COVID‐19) in adults.^30^ In that study, the formulation of either 0.1 or 0.2 ml was nebulized using Aerogen Ultra Device (Aerogen, Galway, Ireland) and was administered via a mouthpiece for 30–60 s. The nebulized aerosols were around 5.4 μm. In the control groups, a dose of either 0.5 or 1.0 ml was injected into the arm's deltoid muscle. They reported a good tolerance to the aerosolized Ad5‐nCoV. Similar antibody responses were induced by two doses of aerosolized Ad5‐nCoV (28 days apart) to one intramuscularly administered dose. A combination of aerosolized and intramuscular vaccinations was also tested. An inhaled booster 28 days after the first injection was observed to elicit strong IgG and antibody response.

There are also other nasal spray formulations for COVID‐19 management and therapeutics that are either before clinical trials or do not have a COVID‐19 (S) protein. These include (1) Xlear nasal spray that contains grapefruit seed extract (GSE) to kill SARS‐CoV‐2 virus and xylitol as a virus decoy,^31^ (2) a composite‐based spray that contains algae‐isolated carrageenan and a gellan polysaccharide studied at the University of Birmingham,^32^ and (3) an aerosolized formulation termed AeroNabs that contains engineered nanobodies similar to those in llamas and camels to impede spike–ACE2 interactions.^33^

More nasal spray formulations are still under active investigation with animal trials. De Vries et al. administered the fusion inhibitory lipopeptide to ferrets intranasally and reported that the spray completely inhibited the transmission of SARS‐CoV‐2 during 24 h among ferrets living with infected peers.^34^ They also suggested that the lipoprotein can be made as a freeze‐dried white powder that does not require refrigeration. Doctors or pharmacists can mix the powder with either sugar or water to make the nasal spray. In contrast, monoclonal antibody therapy is generally more expensive to produce and requires refrigeration for storage.

Frank et al. tested the viricidal efficacy of a nasal spray with povidone‐iodine as an active pharmaceutical ingredient (API) against the SARS‐CoV‐2 virus.^35^ They observed rapid viral inactivation at a contact time of 15 s with a povidone‐iodine concentration as low as 1.25%.^35^ The spray provides an additional layer of protection for up to 4 h, with additional benefits of reducing infectious viral titers and speeding viral clearance, which is promising in mitigating COVID‐19 community transmission as a supplement to personal protective equipment.

Hassan et al. observed that a single intranasal dose of ChAd‐SARS‐CoV‐2‐S (chimpanzee adenovirus‐vectored vaccine encoded with a prefusion stabilized spike protein) in mice could elicit high levels of antibody expression.^28^ This could further promote responses of T cell and mucosal immunoglobulin A (IgA), thus preventing SARS‐CoV‐2 invasion and replication in both the nasal airway and alveolar region.^28^

King et al. tested the efficacy of adenovirus type 5 vectored vaccine against SARS‐CoV‐2 in mice and observed a high level of immune response after a single nasal spray dose both site‐specifically and systemically.^36^ They attributed this high‐level response to the complementary release of serum neutralizing antibodies, mucosal IgA, as well as CD4^+^ and CD8^+^ T cells.

Sun et al. demonstrated that the Newcastle disease virus (NDV) vector vaccine is capable of inducing intensified antibody expression in laboratory mice when given intramuscularly.^37^ The NDV can replicate in humans but is harmless. Nasal spray formulations containing NDV have been suggested to prevent individuals from shedding the virus and developing infections elsewhere in the body. Specifically, engineered NDV can be exploited to produce the SARS‐CoV‐2 spike proteins in order to prime the body's immune system.

### 3.3 Responses of virus variants to therapeutics

Mounting evidence indicates that current vaccines may start to lag behind the fast‐evolving variants, by either increasing affinity to cell receptors, evading neutralizing antibodies, or becoming more refractory to therapeutics.^38^, ^39^, ^40^, ^41^ The new B.1.1.7 variant discovered in the UK exhibits significantly higher infectivity than its ancestors.^42^ It is 70% more transmissible than previous SARS‐CoV‐2 strains. Some of the new variants may become unresponsive to current vaccines or more refractory to existing drugs. In a recent study just published in Nature (March), Wang et al. examined the antibody resistance of two variants: B.1.1.7 discovered in the UK and B.1.351 discovered in South Africa.^43^ They showed that B.1.1.7 is resistant to most monoclonal antibodies at the spike protein's NTD. It also exhibits relatively high resistance to many antibodies at the RBD. With an E484K mutation, B.1.351 shows even higher antibody resistance at both NTD and RBD and is 9.4‐fold more refractory to neutralization than the wild‐type SARS‐CoV‐2, jeopardizing the efficiency of current vaccines and presenting new challenges for vaccine development.^43^ People who had been vaccinated (AstraZeneca and Pfizer in this study) for more than 3 weeks can still test positive, as reported in a new study to appear in Nature (April 30, 2021).^44^

## 4 DEVICES FOR NASAL ADMINISTRATION

### 4.1 ACE2 distribution: where to deliver medicines?

The binding affinity between the S protein and ACE2 is the key factor for SARS‐CoV‐2 infection and symptom severity.^45^ As a result, the physiological body distribution of ACE2 is essential in identifying susceptible organs and tracking disease progression.^46^ ACE2‐expressing respiratory epitheliums are direct targets to invading viruses, potentially giving rise to varying levels of pathological manifestations (Figure 2A). On the other hand, inhalation drug delivery can target the APIs to these regions to either block the receptors, kill the virus, or cure viral damages (Figure 2B).

SARS‐CoV‐2 entry receptors are highly populated in nasal epithelial cells.^13^, ^47^ Lee et al.^48^ investigated the ACE2 subcellular localization within the nose of human donors and reported the highest ACE2 expression within the ciliated epithelial cells, followed by the secretary (goblet/club) cells and suprabasal cells. The nasal goblet cell is one of the three confirmed entry sites of SARS‐CoV‐2, where both necessary entry factors, ACE2 and TMPRSS2 (Type II transmembrane serine protease), coexist.^47^ The other two sites are the alveoli and ileal absorptive cells in the small intestine and all three exhibit higher‐than‐normal viral replication and manifest definitive symptoms during SARS‐CoV‐2 infection. For instance, the loss of smell is commonly experienced in COVID‐19 patients, which coincides with the moderate/high expression of ACE2 in human olfactory mucosa.^49^

Histologically, stratified squamous epithelium lines the anterior one‐third of the nasal cavity, while the columnar ciliated epithelium, mucus‐producing goblet cells, and suprabasal membrane line the posterior two‐thirds of the nasal cavity and nasopharynx.^50^ The exceedingly high ACE2 expression in the back nose also explains why nasopharyngeal swab specimens have been predominantly used in COVID‐19 testing for early virus detection.^51^ This also means that the nasally administered vaccines should be delivered to the posterior two‐thirds of the nose to be effective to interact with ACE2 (Figure 2A). A device that can effectively dispense the vaccine to the target for the optimal outcome is crucial considering the shortage of vaccines.

### 4.2 Nasal sprays

**FIGURE 3:**
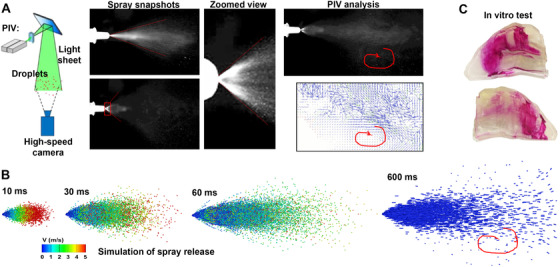
Nasal spray characterization, in vitro testing, and numerical simulations: (A) Sar‐Gel visualization of nasal spray deposition, reprinted with permission.^53^ (Copyright 2016, Springer Nature), (B) stereo‐PIV characterization of the exiting speed, droplet diameter, and plume angle, and (C) mathematical model for nasal spray release (with a polydisperse droplet distribution, a normal distribution of exiting speeds, and a prescribed plume angle) and computationally predicted spray distribution using an Eulerian wall film model at two instants after spray injection

Nasal spray devices have long been recommended to deliver influenza vaccines.^52^ However, the majority of nasal spray droplets will deposit in the anterior nose, including the nasal vestibule and nasal valve, while only a small portion can reach the middle turbinate region.^53^ Figure 3A shows a typical distribution of nasal sprays in a patient‐specific nose cast. The nose cast was split along the ridge of the right nasal passage to disclose the deposition pattern on both the turbinate region and nasal septum. The low delivery efficiency is attributed to both the nasal spray properties and nasal physiology, as described below.

Parameters of the nasal spray devices that affect the delivered dose distribution include the droplet size distribution, droplet exiting speed, spray plume angle, nozzle orientation relative to the patient's nostril, and applied dosage. The nasal spray droplets are generally large, ranging from 20 to 300 μm in diameter and with inertia impaction being the predominant deposition mechanism.^54^ Using nasal cast replicas, Cheng et al. measured the deposition distributions with different nasal spray pumps.^55^ It was observed that elevated deposition in the front nose was associated with large droplets and wide plume angles, while smaller droplets and narrow plume angles improved turbinate dosimetry. Kundoor and Dalby evaluated the device orientation effect from 0° to 90° in 15° increments.^56^ It was concluded that an angle of 60° from the vertical direction delivered the most favorable dose in the nasal cavity. Even for a given device and administration orientation, the drug distribution can vary when different dosages of nasal sprays are applied because of the liquid film translocation.^57^

To accurately predict nasal spray dosimetry, key parameters should be quantified. The size distribution of the nasal spray droplets can be measured using laser diffraction. The spray plume angle and droplet exiting speeds can be estimated using a stereo‐particle imaging velocimetry (PIV) system. Figure 3B shows the images of the droplet motion captured by a high‐speed camera at 2000 frames per second for 2 s. It was observed that the spray process is highly variable during the administration, with varying plume angles and mass flow rates (Figure 3B). The spray plume angle was observed to range from 45^o^ to 90^o^. Short durations of ∼0.1 s were segmented and used for PIV analysis to quantify the droplet speeds. Due to the coalescence, breakup, and evaporation of the droplets, it is difficult for PIV to keep track of individual droplets in order to calculate the droplet speeds; therefore, only high‐quality images within a short duration can be used for this purpose. The calculated speed was estimated to be around 8.5±3.5 m/s, which could be implemented for subsequent numerical simulations.

**FIGURE 4:**
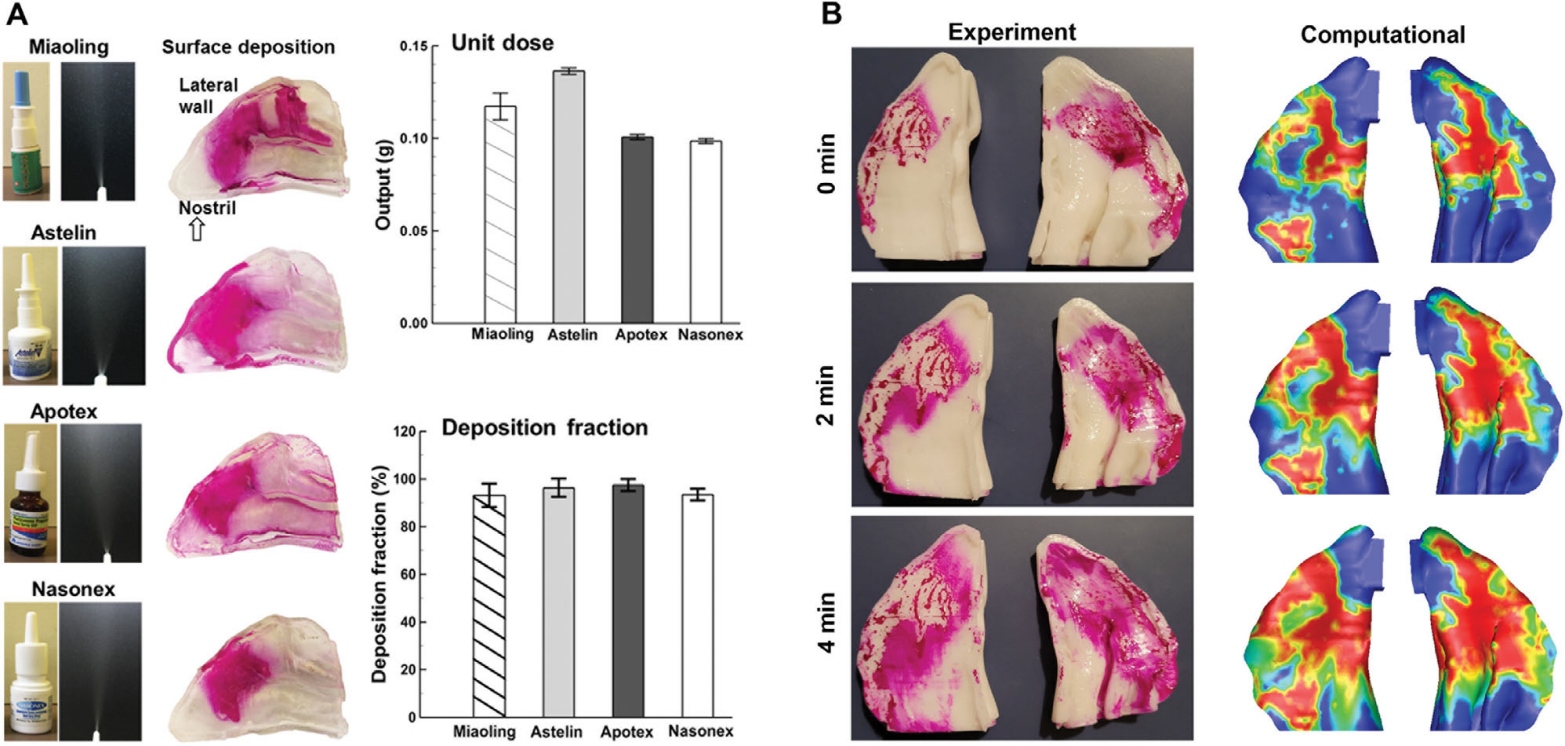
Nasal sprays: (A) in vitro tests of nasal sprays: plumes, deposition patterns, and doses in the nose cast using different nasal spray products, reprinted with permission.^53^ (Copyright 2016, Springer Nature), (B) translocations of deposited sprays: experiments versus computational results at 0, 2, and 4 min after nasal spray application

Our previous in vitro tests confirmed the dominant depositions of nasal spray droplets in the front nose.^53^, ^58^ Figure 4A visualizes the deposition of nasal sprays using Sar‐Gel.^53^ The plume angle of the sprays was 19^o^±0.6^o^, 35^o^±0.8^o^, 33^o^±0.8^o^, and 20^o^±0.5° for Miaoling, Astelin, Apotex, and Nasonex, respectively. It was observed that most spray droplets were deposited in the front nose, particularly around the flow‐limiting nasal valve. The unit output (per stroke) of the nasal sprays was 0.12±0.15 g. Close to 100% of nasal sprays administered into the nostril(s) deposited inside the nose (right lower panel, Figure 4A).

Translocation of deposited spray droplets is another factor that needs consideration. Liquid film formation and dripping in the nose were also observed due to gravity in both in vitro testing and numerical simulations. Figure 4B shows the temporal variation of the drug distribution visualized using Sar‐Gel at 0, 2, and 4 min after applying the nasal sprays. The nasal cast had a supine position. It is demonstrated that the deposited liquid formulation expanded, especially along the gravitational direction. This is reasonable considering that the liquid film sticks to the wall due to a force balance between the surface tension and gravity. Our knowledge of the transient deposition pattern for nasal sprays, however, is still limited at this moment.^57^ Further investigations are needed to evaluate the liquid film formation and translocations for different sprays and with different doses.

Other properties of the nasal spray droplets include electrostatic charges, hygroscopic effect (condensation and evaporation), and droplet interactions (coalesce and breakup).^59^, ^60^, ^61^ The electrostatic force between positively charged droplets and the negatively charged nasal mucus can notedly affect the deposition of small, low‐speed droplets, but not large, high‐speed droplets with large inertia.^62^ Conversely, the electric guidance of charged particles can be used to target aerosols to the desired regions.^63^, ^64^, ^65^ The liquid droplet can change its size due to evaporation or condensation depending on the local ambient relative humidity, leading to a dynamic interplay with the local airflows that will be different from constant‐diameter particles.^61^, ^66^ Depending on the relative velocity between the airflow, droplet breakup can occur.^67^ Similarly, depending on the local droplet concentration, the aerosol cloud effect can change the bulk behavior of the droplet and its subsequent deposition.^68^

### 4.3 Nebulizers

**FIGURE 5:**
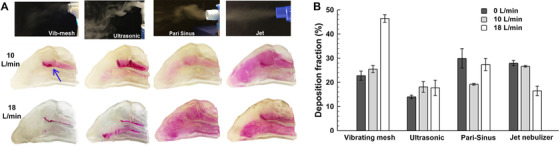
In vitro tests of different types of nebulizers (i.e., vibrating mesh, ultrasound, Pari Sinus, and jet nebulizer): (A) soft mists and nasal deposition patterns, and (B) deposition rates at different inhalation flow rates. Reprinted with permission.^53^ (Copyright 2016, Springer Nature)

The dosage form of nasally administered products can significantly affect their delivery efficiency and distribution in the nose. Nebulizers have also been utilized for intranasal delivery. Depending on the aerosol generation techniques, there are three main categories: vibrating mesh nebulizer, ultrasonic nebulizer, and jet nebulizer. Figure 5A visualizes the nebulized aerosol distribution in the nasal casts using four nebulizers (i.e., Drive Voyager Pro mesh nebulizer, Respironics Ultrasound nebulizer, Pari Sinus nebulizer, and Philips Respironics jet nebulizer). Utilizing a vibrating mesh to generate aerosols, the Drive Voyager Pro nebulizer produces more homogenous droplets than other types of nebulizers. By contrast, the droplets generated by the Respironics Ultrasound nebulizer are larger and more heterogeneous in size distribution. The Philips Respironics Essence is a classic jet nebulizer and generates aerosol droplets at relatively high speeds. Pari Sinus also utilizes the jet atomization technique but generates droplets with much lower speeds. Moreover, an auxiliary flow with an oscillating frequency of 45 Hz and an oscillating amplitude of 24 mbar is implemented to facilitating the delivery to paranasal sinuses. From Figure 5A, significantly different nasal deposition patterns are noted among the four nebulizers. Furthermore, the inhalation flow rates also affect the deposition distributions.

Low‐speed soft mists (or aerosol clouds) are noted in all nebulizers except for the jet nebulizer, which exhibits an apparent jet flow (upper right panel, Figure 5A). Downward droplet motions occurred after discharging from the ultrasonic nebulizer due to the lower speeds and larger droplet sizes. The deposition patterns were remarkably different among the four nebulizers (middle panel, Figure 5A), from focused (mesh nebulizer) to wide‐spread (jet‐type) patterns. At a low inflation rate (10 L/min), there was a streak of deposited sprays along the edge of the middle turbinate (blue arrow), which was consistent with the main inspiratory flow. At a higher inhalation flow rate of 18 L/min, more aerosols are transported to the median nasal passage and reduce the deposition on the turbinate edge. Considering the ultrasonic nebulizer, similar patterns were obtained at 10 and 18 L/min. Considering the Pari Sinus, the deposition distribution varied from less diffusive at 10 L/min to more widespread at 18 L/min. In the jet nebulizer, the highly dispersed deposition was found at both flow rates. Also, there was less deposition in the upper nose at 18 L/min. The core flow mainly occurred in the lower and median passages and entrained aerosols that otherwise went to the upper nose.

The deposition rates of nebulized aerosols are shown in Figure 5B at three respiration flow rates, with each test case being repeated five times. Comparing to nasal spray products, the deposition of nebulized aerosols is much lower. The maximum DF is 46% for the mesh nebulizer at 18 L/min and the minimum DF is 15% for the ultrasonic nebulizer at 0 L/min (breath‐holding). Interesting trends are noted in the DF variation with flow rate for different nebulizers: the DF increases with the respiration rate in the mesh and ultrasonic nebulizers, while it decreases with the respiration rate in Pari Sinus and Philips jet nebulizers.

### 4.4 Delivery methods: bidirectional versus normal

**FIGURE 6:**
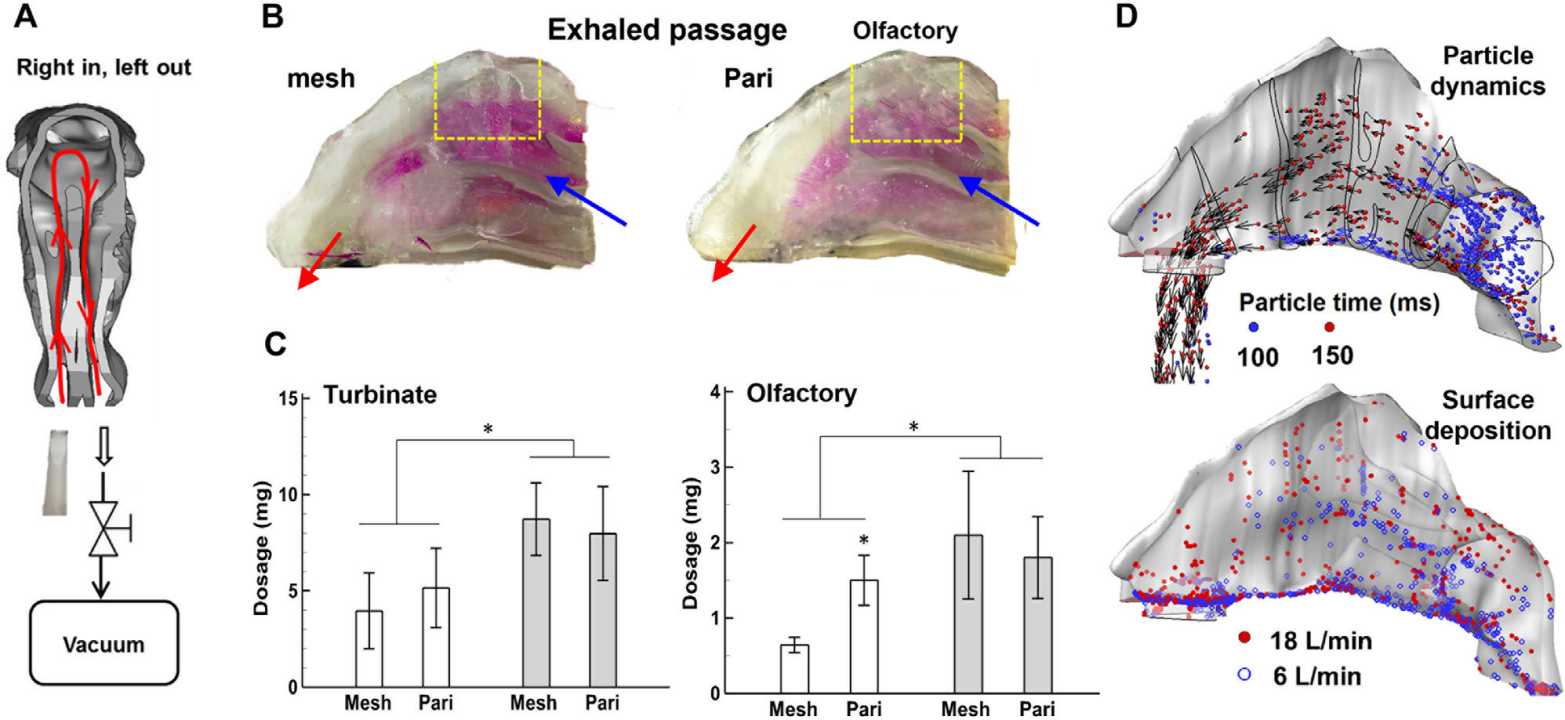
Deposition pattern in the second (exiting) nasal passage using the bidirectional delivery protocol for the mesh (Voyager Pro) and PARI Sinus nebulizers: (A) delivery diagram (right in, left out), (B) deposition pattern, (C) deposition fractions in the turbinate and olfactory region, and (D) simulation results of the particle dynamics and deposition distribution in the exhaled passage. Reprinted with permission^72^

Unwanted drug loss into the lungs can be another issue for nasal drug delivery. With standard intranasal delivery methods, significant portions of drugs can enter the lungs with inhalation air flows if particles are small and inertial depositions are limited. During inhalation, the nasopharynx remains open as the rear portion of the soft palate flips downward. The unwanted lung dosages can adversely affect both the lungs and liver as a result of entering the bloodstream through the lungs. To address this issue, Djupesland et al. tested an alternative method named bidirectional delivery technique.^69^ This technique took advantage of the nasopharynx closure during exhalation due to the lift up of the soft palate and therefore, close the entrance to the lung from the nose. A dose of medicine is administered into the nose by having the patient blow into the delivery unit to trigger the release of drug particles.^70^ Because the soft palate blocks the nasal cavity from the mouth during drug delivery, particles will first enter one nose, sequentially travel through the two nasal passages by taking a U‐turn at the nasopharynx, and exit through the other naris (Figure 6A). This process gives the drug particles more time in the nose and possibly enlarges the nasal cavity due to higher flow resistance. More importantly, the uplifted soft palate restricts the particles within the nose, thereby eliminating the issue of lung contamination. It has been demonstrated that the bidirectional delivery system reduced the dripping in the nose and increased drug deposition at the top portion of the nasal cavity in comparison to standard nasal devices, such as dry powder inhalers (DPIs) and nebulizers.^71^

Different patterns of particle deposition are expected in the two passages under the bidirectional breathing pattern.^72^ To visualize the deposition pattern in the second passage, the direction of the bidirectional delivery was reversed, namely, from “left in right out” to “right in left out” (Figure 6A), so that particle deposition in the left (exiting) passage could be revealed. As expected, much fewer aerosols were deposited in the second passage. Figure 6B shows the deposition pattern after 1 min's administration. This diminished deposition is reasonable considering that the majority of the administered droplets have been filtered out in the first (entrance) passage. Overall, the bidirectional technique yielded higher depositions in both the nasal cavity and olfactory region compared to the normal technique. It is interesting to see from Figure 6C that PARI Sinus performs better with the normal delivery method, while the mesh nebulizer is better with the bidirectional technique. This trend is valid in both the nasal passages and olfactory region.

To further understand the bidirectional effects on particle behaviors, snapshots of particle motion at different time points after releasing were computed (Figure 6D). The velocity vectors were also plotted on particles at selected instants. After 100 ms, particles approached the nasopharynx and changed directions sharply in the bidirectional mode to enter the second passage, leading to greater resistance than the normal mode. This increased resistance would affect the airflow and particle dynamics in both nasal passages, and the second passages in particular due to the ambient pressure at its exit. The diffusive surface deposition predicted by numerical simulations (lower panel, Figure 6D) is consistent with that obtained via in vitro tests (Figure 6B).

### 4.5 Other intranasal delivery options

Other delivery devices via the nasal route include the soft‐mist inhaler, pMDI, and pMDI with a holder chamber and/or face mask.^73^ These devices, however, are meant for pulmonary drug delivery rather than to the nose. A DPI needs the patient's oral inhalation for actuation and is not suited for nasal delivery.^74^

Inhalers used for pulmonary drug delivery need more precautions than nasal spray devices when used in COVID‐19 patients, because of the higher chances of virus‐laden droplets exhaled from the deep lungs.^20^ Studies show that the above three solution‐based inhalers differ in their safety during device preparation and COVID‐19 inhalation therapies. The lowest contamination and viral transmission were found in the soft‐mist inhaler; however, patient training was needed to achieve accurate hand‐breath coordination.^75^ pMDIs are also shown to have a low risk for viral transmission due to their short treatment time and low emitted dose.^76^ The safety data of nebulizers with COVID‐19 patients are still controversial in light of solution filling and aerosol dispersion versus passive inhalation.^73^

### 4.6 Safety issues during spray administration

Aside from inflicting the patients, the SARS‐CoV‐2 virus also poses a serious risk to the healthcare workers (HCWs) who treat them. Exposure of the HCW to the patients' exhaled aerosols and during aerosol treatments is a concerning issue when performing inhalation therapies. Insufficient abidance to the safety procedures can increase the chance of infection in the working places.^77^, ^78^ To minimize occupational transmissions, infection control measures should be strictly followed. Hui et al.^79^ investigated exhaled leakage jet plume from the nebulizer facemask applied to an adult patient simulator that was programmed at both normal and diseased conditions, the latter with different levels of respiratory injury. They observed that the aerosol dispersion distances mainly depended on the aerosol‐generating procedures to treat lung injuries at different severities. The maximum aerosol dispersion distance was measured to be 0.45 m at the normal lung condition, 0.54 m at the mild condition, and 0.8 m at the severe condition.^79^ A safety distance of 0.8 m or more was recommended for HCWs being apart from patients receiving nebulizer treatments. Protective facemasks are always recommended to patients who are suspected or confirmed to have contracted COVID‐19. Full protective equipment, including surgical respirators type N‐95, gowns, double gloving, and frequent hand washing, were recommended for the HCWs all‐time during the inhalation therapy.^19^ Further studies are needed to quantify the aerosol dispersion distance from different inhalation devices used for COVID‐19 management and evaluate the risks of viral transmission due to the aerosol dispersions from respective devices.

## 5 NASAL PHYSIOLOGICAL FACTORS

### 5.1 Uniqueness of human nasal passage

The nasal cavity has two relatively symmetric passages divided by the septum. The nasal passage starts from a wedge‐shaped or oval nostril, which is open to a curved atrium called the vestibule. Moving forward, the vestibule quickly becomes narrow and converges into a slit‐like nasal valve, which is the most constricted and distensible structure in the nose. The nasal valve collapses when the inhalation rate exceeds 34 L/min and thus acts as a flow‐limiting switch (or valve to oral breathing).^80^ After the nasal valve, the nasal passage quickly expands into a narrow and convoluted channel featuring three fin‐like curved airspaces termed the inferior, middle, and superior meatus from bottom to top, respectively. The convoluted boney structure below the respective meatus is called the inferior, middle, and superior conchae or turbinate, which together act as the sidewall for the nasal cavity. The two nasal passages merge at the posterior nose, which expands to the nasopharynx and leads to the throat and lung.

The labyrinth structure of the nasal cavity generates unique respiratory airflows, which facilitates it to realize specific physiological functions, such as warming and moistening inhaled air and cleansing inhaled particles.^81^, ^82^ On the other hand, nose function deficiency often results from the deviated nasal structure and modified aerodynamics inside it.^83^, ^84^, ^85^, ^86^, ^87^ The flow‐limiting slit‐like nasal valve intercepts most of the spray droplets that possess high inertia and cannot follow the convergent accelerating streamlines toward the nasal valve.^58^ Many might not realize how hard the nose works for the human body on a daily basis. It inhales 18,000–20,000 L of ambient air per day (for a typical adult), primes the air to body temperature and relative humidity, filters out the airborne pollutants to protect the delicate lungs, and recovers the moisture in exhaled air. Beating cilia on the nasal epithelium transport the filtered particles to the throat, which are either coughed out or swallowed. The nose also protects the olfactory nerves by housing them in the superior meatus and provides them an extremely low fraction of inhaled air but with enough chemical molecules for the olfactory nerves to perceive.^88^ The maxillary sinuses generate NO that has an anti‐inflammatory effect and cures airway injuries all the time.^89^

### 5.2 Nasal geometry variation with COVID‐19 infection

Patients infected with SARS‐CoV‐2 often experience nasal dysfunctions, such as nasal congestion, loss of smell, rhinorrhea, and cacosmia.^90^ Because of the high concentration of capillary vessels within the nasal mucosa, variations in these capillaries can lead to nasal congestion (and rhinorrhea) and significantly block the already very narrow nasal passages. This obstruction will further reduce the delivery efficiency to the posterior nose, thereby diminishing the therapeutic efficacy.^58^ To tackle this problem, a physical or chemical dilator should be applied before spray administration. Further studies, either in vivo, in vitro, or in silicon, should be undertaken to quantify the nasal spray dosimetry in congested noses of varying severities. Up to date, quantitative studies in this aspect are still scarce.

The nasal congestion will also block the ostia connecting the sinuses to the main nasal passages. Sinuses generate NO and release it to the nose.^89^, ^91^ As aforementioned, NO has antifungal, antiviral, antibacterial, and anti‐inflammatory effects, and therefore plays an essential part in sustaining the health and functions of the airway. Interference with this endogenous nasal nitric oxide production and release can further aggravate the symptoms.

### 5.3 Intersubject variability: nasal dilation effect

**FIGURE 7:**
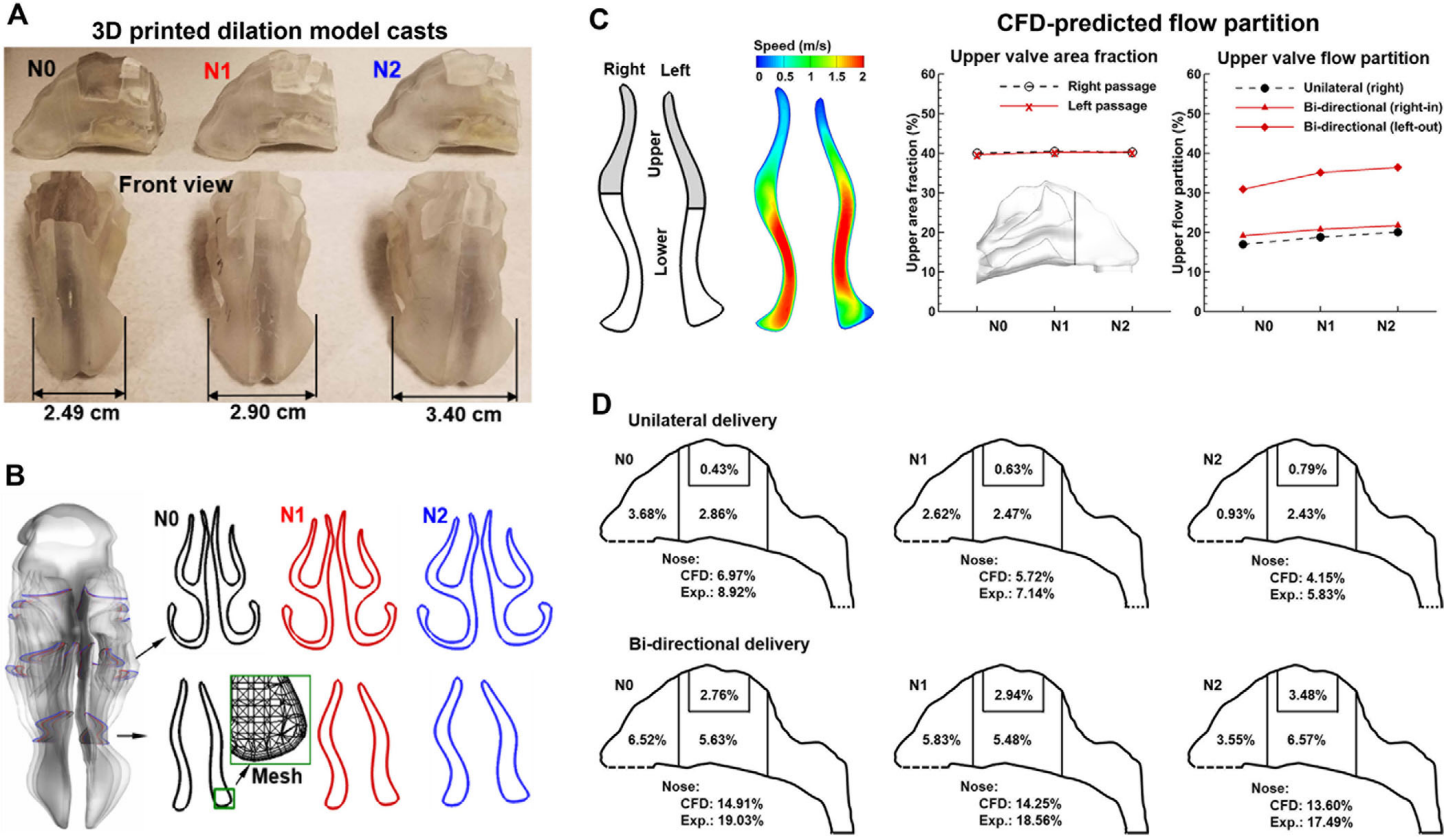
Nasal passage dilation effects: (A) 3D printed casts of three nasal models (N0, N1, and N2) with increasing passage dilations, (B) cross‐sections of the three nose dilatation models at the nasal valve and turbinate, (C) CFD‐predicted flow partition, and (D) comparison of CFD and in vitro measured deposition fractions among the three models under unilateral and bidirectional deliveries. Reprinted with permission^58^

The human nose is a compliant structure that can change its shape/size either passively or actively to adjust airflow ventilation. Nasal expansion can alter the dosimetry of inhaled aerosols within the nasal cavity.^58^, ^92^ To quantify the dosimetry variation from nasal dilations, both in vitro tests and numerical simulations were undertaken in three nasal models (N0, N1, and N2) with progressive dilation in the nasal passages (Figure 7A,B). Specifically, gradual expansion was made to the nasal valve region, as evidenced by the front nose width of 2.49, 2.90, and 3.40 com in N0, N1, and N2, respectively (Figure 7A). Relative to the control case N0, the expansion rate of the nasal valve was 30% and 50% in N1 and N2 (Figure 7B).

Nasal dilation, especially nasal valve dilation, can noticeably affect flow partition in the nose. Both nasal valves were split into the upper and lower zones and their ventilation rates were quantified (Figure 7C). In contrast to a similar upper‐lower area ratio (∼39%) for the three models, the flow ventilation to the upper zone was much lower, that is, 17–22%. Moreover, valve dilation enhanced the flow partition to the upper zone, which was 17% in N0 and 20% in N2 for the normal (unilateral) delivery and was 18.5% in N0 and 21% in N2 for the bidirectional delivery. The flow partition to the upper valve increased significantly in the left nose (the exhalation nasal passage) for the bidirectional delivery, with a 30.5% flow partition in N0 and 36% in N2. In comparison to the right nose (inspiratory nasal passage), the high‐speed flow zone in the left valve shifted upward, as demonstrated by the speed contour in Figure 7C.

Numerically predicted deposition fractions (DFs) are shown in Figure 7D for the three models in comparison to in vitro measurements. Good agreement was obtained between the measured and predicted DFs both in magnitude and trend. The predicted DFs slightly, but consistently, underestimated the experimental data. For both delivery methods, the vestibular dosage decreased with the valve dilation. Specifically, a large decrease in the vestibular dosage occurred from N1 to N2 with the normal delivery method. On the other hand, the olfactory dosage increased with the valve dilation for all cases herein, which was 0.43%, 0.63%, and 0.79% in N0, N1, and N2, respectively. Further increased olfactory DFs were achieved using the bidirectional method, with the olfactory DF being 2.76%, 2.94%, and 3.48% in N0, N1, and N2, respectively. The optimal olfactory DF with bidirectional delivery (i.e., 3.48% in N2) was around three times higher than the unilateral olfactory DF in N2 (0.79%) and seven times higher than that in N0 (0.43%).

### 5.4 Intersubject variability: age effect

Another unfortunate consequence of this pandemic is the age‐dependent susceptibility and mortality rate, where older ages appear to directly associate higher risks and more severe outcomes.^93^ Bunyavanich et al. compared the nasal epithelial ACE2 gene expressions between young children and adults in a cohort of 305 subjects ranging from 4 to 60 years old.^94^ They reported that the lowest ACE2 expression (2.40, mean log_2_ counts per million) occurred in young children (*n*  =  45) and that the ACE2 expression increased with age, with the value being 2.77 in older children (*n*  =  185), 3.02 in young adults (*n*  =  46), and 3.09 in senior adults (*n*  =  29).^94^ The significantly lower *ACE2* expression in children may attribute to the lower COVID‐19 cases in this age group.

Studies also showed an elevated level of expression of *ACE2* and *TMPRSS2* in the respiratory tissues of smokers and COPD patients.^95^ By contrast, a lower level of ACE2 expression was observed in asthmatic and allergic patients, which may lower their infection risks and symptom severities.^96^

**FIGURE 8:**
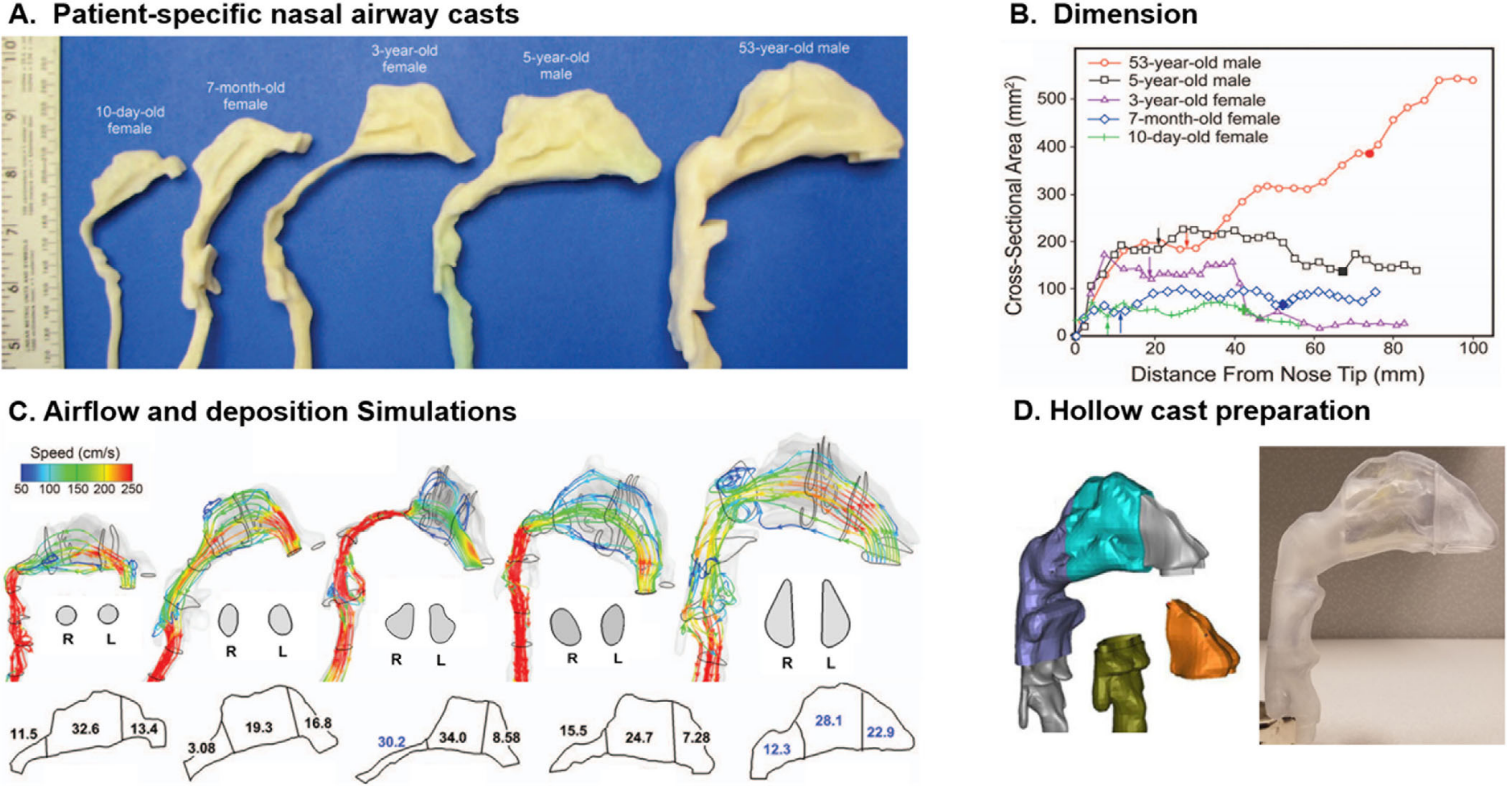
Age‐dependent variability: (A) patient‐specific nasal geometries reconstructed from CT scans: 10‐day‐old female, 7‐month‐old female, 3‐year‐old female, 5‐year‐old male, and 53‐year‐old male, (B) age‐dependent airway dimension, (C) airflow and 10‐μm aerosol deposition, and (D) 3D printed hollow cast for in vitro testing. Reprinted with permission^97^

The human nose progressively changes its shape and size from neonate to adult (Figure 8A), which significantly alters the dosimetry and distribution of applied nasal sprays (Figure 8B).^97^, ^98^, ^99^, ^100^ The normal body weight ranges from 2.5 kg for a newborn to 80 kg for an adult, with a scale factor spectrum of 32. Generally, the nostrils of a younger subject are more circular.^101^, ^102^, ^103^ His/her turbinate is also less complex, indicating undeveloped bones and tissues in young children, as shown in Figure 8A. This difference has an important implication on nasal spray applications, as the spray dosage is highly sensitive to the nasal geometrical complexities.^53^, ^58^ Due to nonproportionally large tonsils and adenoids, the nasopharynx in children appears to be much more slender than that in adults, which is also the key factor behind the prevalence of snoring in children between 2 and 3 years old.^104^ Furthermore, the pharynx is also thinner in children than the adults. How these age‐dependent morphological variations affect the nasal spray dosimetry warrants further numerical and in vitro studies (Figure 8C,D). Such a need is becoming increasingly urgent as the SARS‐CoV‐2 variants have caused more hospital admissions and serious COVID‐19 cases in children and young people, while the world is still witnessing outbreak waves afflicted by constantly emerging SARS‐CoV‐2 mutants in different countries.^105^, ^106^

### 5.5 Nasal mucociliary clearance

Mucociliary clearance in the nose should be considered in the development of nasal spray therapeutics and vaccines, especially those that are intended for long‐acting protection.^107^, ^108^ The cilia on the nasal epithelium move the mucus upon it toward the throat at a rate of 8–10 mm/h.^109^, ^110^ In doing so, they protect the airway from injury by deposited agents from the inhaled air. However, the mucociliary clearance also removes the vaccine from the active site within 1–3 h considering the posterior two‐thirds of the nose is approximately 30 mm in length for a typical adult.^111^ Pharmacokinetic calculations also show that nasal mucociliary clearance can cause a significant discrepancy in drug absorption and drug bioavailability following intranasal administration.^112^, ^113^ The beat frequency of the cilia is 12–15 Hz under normal conditions and its variation has been used as an index to assess drug toxicity.^114^ Both the cilia beat frequency and the mucus secretion increase when challenged with drug formulations, which quickly dilute the formulation and transport it to the throat. The mechanism shortens the resident time of the drug formulations, which reduces the drug bioavailability and presumably decreases therapeutic outcomes.^115^

To ensure the adequate bioavailability of nasally inhaled vaccines and therapeutics, absorption enhancers can be implemented, like phospholipids, cyclodextrins, bioadhesive powders, and chitosan.^116^ In particular, chitosan, a sugar from the outer skeleton of shellfish, is a safe and effective absorption enhancer for nasal products, such as peptides, proteins, small hydrophilic drugs, polysaccharides, and nucleic acids, which otherwise cannot translocate through the mucus layer.^117^, ^118^ The safety issues related to absorption enhancers should be evaluated, such as their toxicity, mucosa irritability, and airway injury.^119^ Previous studies have shown that chitosan has low nasal toxicity and high mucosa compatibility, which supports its use as an absorption enhancer in nasal spray formulations.^120^

## 6 SUMMARY

In response to the unmet need for effective control of SRAS‐Cov‐2 transmission, research efforts of inhaled therapy, including nasal sprays, have been actively undertaken since the pandemic outbreak. Nasal sprays can be an alternative and additional intervention to prevent COVID‐19 transmission as the first‐line defense to the virus. There are many advantages of a nasal spray vaccine or therapeutics, including it being noninvasive, triggering local immunity, reducing systemic side effects, and feasible to be self‐administered.

Nasally inhaled medicines include repurposed or antiviral drugs. The latest development of such nasal sprays was presented, covering those currently undergoing clinical trials, those that are still in the stage of preclinical or animal studies, and those as a prophylactic or as a vaccine. Underlying antiviral mechanisms for each formulation were presented in hope that expected therapeutic outcomes (and efficacy) can be estimated.

Factors that influence the spray dosimetry and distribution in the nose were discussed in detail. These included the target sites (regions of high ACE2 expression), the nasal devices, spray characterization, and safety issues during therapy. Drug waste in the anterior nose was associated with large droplets and wide plume angles, while effective delivery of vaccines to the posterior nose necessitates small droplets and narrow plume angels with a device orientation of about 60^o^. A safety distance of 0.8 m or more is recommended between HCWs and patients receiving inhalation therapies. Human nasal physiological factors that regulate nasal spray dosimetry were listed, including normal nasal geometry, geometrical modification with COVID‐19 or other respiratory diseases, age‐related intersubject variability, and mucociliary clearance.

Nasal inhaled therapeutics and vaccination can be a potent alternative for COVID‐19 therapeutics and management in the developing world. Research to develop low‐cost, easy‐to‐use nasal spray vaccines with readily available materials and facilities is encouraged to this aim. Nasal spray vaccines based on traditional and complementary medicines, such as GSE, algae‐isolated carrageenan, and Yogurt‐fermenting *Lactobacillus*, are promising and under active development. There is a compelling need to develop an effective delivery system to target formulations at the ACE2‐rich regions to achieve optimal outcomes for both adults and children, health (as a prophylactic), and disease (as a therapeutics). This need is particularly pressing in a context that emerging SARS‐CoV‐2 variants may evade current vaccines and develop resistance to existing therapies. This need is more real than ever by affecting each one of us considering the increasing confirmed cases in children and the devastating outbreak waves in India and many other countries around the world.

## CONFLICT OF INTEREST

The authors declare that they have no conflict of interest.

## ETHICS APPROVAL

The usage of medical images in the development of airway models was approved by the Institutional Review Board of the University of Massachusetts, Lowell.

## AUTHOR CONTRIBUTIONS

J.X., L.L. and X.S. conceived the project and designed experiments. J.X., L.L., W.Z. and X.S. conducted the literature review. J.X. X.S. and W.Z. performed experiments. J.X., L.L. W.Z. and X.S. analyzed the data. J.X. and X.S. wrote the manuscript.

## Data Availability

The data used in this review are available from the corresponding author at reasonable request.
